# Relationship between phenotypic subcutaneous backfat thickness and spermiogram outcomes in young beef bulls

**DOI:** 10.1093/tas/txaf039

**Published:** 2025-04-04

**Authors:** Molly S Smith, Jorge Octavio Solano Aguilar, Grace Nyhuis, Francine Messias Ciriaco, Saulo M Zoca, Lew Strickland, R Lawton Stewart, Jason D Duggin, Pedro Levy Piza Fontes

**Affiliations:** University of Georgia, Department of Animal and Dairy Sciences, Athens, GA 30602, USA; University of Georgia, Department of Animal and Dairy Sciences, Athens, GA 30602, USA; University of Georgia, Department of Animal and Dairy Sciences, Athens, GA 30602, USA; University of Georgia, Department of Animal and Dairy Sciences, Athens, GA 30602, USA; University of Tennessee, Department of Animal Science, Knoxville, TN 37996, USA; University of Tennessee, Department of Animal Science, Knoxville, TN 37996, USA; University of Georgia, Department of Animal and Dairy Sciences, Athens, GA 30602, USA; University of Georgia, Department of Animal and Dairy Sciences, Athens, GA 30602, USA; University of Georgia, Department of Animal and Dairy Sciences, Athens, GA 30602, USA

**Keywords:** Backfat, Beef, Bull, Fertility, Semen, Sperm

## Abstract

This study investigated the relationship between subcutaneous backfat thickness (SCBF) and semen quality in young beef bulls. We hypothesized that bulls with increased SCBF would have decreased semen quality compared with bulls of adequate SCBF, despite being fed the same diet. Data collected from yearling beef bulls (*n* = 710) enrolled in two bull development programs were utilized in an observational retrospective cohort study. Bulls were developed according to industry standard practices and growth performance was evaluated over 84 or 112 d following a 14- or 21-d adaptation period. At the end of the growth performance evaluation period, carcass ultrasonography was performed to evaluate ribeye area (REA), SCBF, and intramuscular fat. Furthermore, breeding soundness examinations (BSE) were performed within 40 d after carcass ultrasonography. Bulls that failed the BSE for reasons unrelated to semen quality were excluded from this study. To evaluate the relationship between SCBF and fertility variables, bulls were categorized into three cohorts based on SCBF estimates using two distinct thresholds. Within each location and year, bulls were retrospectively ranked according to their SCBF and classified into the top 10% (TOP10; n = 71), middle 80% (MID80; n = 569), and bottom 10% (BTM10; n = 70). To further investigate the relationship between SCBF and fertility, bulls were also categorized into the top 20% (TOP20; n = 153), middle 60% (MID60; n = 419), and bottom 20% (BTM20; n = 138) based on SCBF. TOP10 and TOP20 bulls had greater initial and final body weight, SCBF, REA, and IMF (*P* ≤ 0.01) compared with their respective cohorts. In contrast, SCBF classifications did not impact average daily gain, scrotal circumference, and sperm motility (*P* ≥ 0.29). The percentage of morphologically normal sperm was decreased in TOP10 and TOP20 bulls compared with their respective cohorts (*P *< 0.01), which are similar among them (*P *≥ 0.31). These differences resulted in a greater proportion of TOP10 (*P* < 0.01) and TOP20 (*P* < 0.01) bulls classified as deferred compared with their respective cohorts. In summary, elevated SCBF in bulls exposed to the same diet was associated with an increase in sperm morphological abnormalities and resulted in a larger proportion of bulls classified as deferred during their first BSE.

## Teaser

Increased adiposity is negatively associated with semen quality in yearling beef bulls.

## INTRODUCTION

Reproductive efficiency plays a crucial role in the profitability of cattle operations, with reproductive failures costing the United States cattle industry over $2 billion annually (Lamb et al., 2016). Historically, research in bovine reproductive physiology has predominantly centered on female fertility, likely because key reproductive performance indicators in cattle (e.g., pregnancy rates, calving intervals, and age at first calving) are evaluated in females. While research has largely been focused on improving female reproductive efficiency, the fertility of bulls is significantly important to the overall reproductive success of the herd. Enhancing our knowledge of environmental and management factors influencing bull fertility could significantly improve reproductive efficiency and productivity in cow-calf production systems.

Sire over-conditioning is a phenotype that is commonly observed in the beef industry. Extension programs and bull development stations across the United States have reported the general preference of beef cattle producers for bulls with increased pre- and post-weaning growth characteristics. When selecting herd sires, cattle producers often prioritize growth performance and growth-related genetic traits versus feed efficiency estimates, such as feed-to-gain ratio, residual average daily gain, and residual feed intake (McDonald et al., 2010; Oosthuizen et al., 2018). These preferences result in market signals encouraging seedstock producers to develop bulls with diets that induce rapid growth rates and fat deposition to achieve this desirable phenotype. Therefore, bull development programs often observe rates of body weight gain that resemble what is observed in feedlot steers (Marques et al., 2017; Pancini et al., 2020).

Previous literature indicates that experimentally exposing bulls to high-energy diets negatively impacts semen quality based on traditional estimates of sire fertility (Hopkins and Spitzer, 1997; Koziol and Armstrong, 2018). Several studies reported that sperm motility was reduced and sperm morphology defects were increased when bulls were experimentally fed diets to nutritionally induce an over-conditioned phenotype (Coulter and Kozub, 1984; Coulter et al., 1997). While a detailed description of the impact of highly anabolic diets on sperm biology in the bovine is limited, research in humans and murine models indicates that diet-induced obesity results in increased systemic inflammation, sperm oxidative stress, DNA fragmentation, and epigenetic modifications that collectively impair spermiogram results and reduce fertility (Houfflyn et al., 2018; Barbagallo et al., 2021). Interestingly, these changes in sperm biology induced by highly anabolic diets have been shown to negatively impact early embryonic development after fertilization takes place (Mitchell et al., 2011; Seekford et al., 2023) and might also program post-natal development of the offspring (Grandjean et al., 2015; Chen et al., 2016; Raad et al., 2017).

While it is established that over-conditioning in beef bulls adversely affects semen quality, the impact of natural variation in body fat accumulation on spermiogram results within a population of bulls exposed to the same diet has not been thoroughly investigated. Observational studies are crucial in this context, as they reflect the industry-standard conditions and natural variation that animals experience in typical cattle production settings. Understanding how these naturally occurring differences in fat accumulation influence semen quality provides insights that are not only directly applicable to industry practices but also strengthen the relevance of experimental models to study bull over nutrition. We hypothesized that bulls with excessive subcutaneous backfat thickness (SCBF) exhibit decreased semen quality during industry-standard breeding soundness examination (BSE) compared with bulls having adequate SCBF. Moreover, we hypothesized that these differences in semen characteristics would result in a greater percentage of bulls with excessive SCBF failing the BSE. The objective of this study was, therefore, to examine the relationship between SCBF and spermiogram outcomes in young beef bulls exposed to the same diet and environmental conditions.

## MATERIALS AND METHODS

### Animal Management

This observational retrospective cohort study used records obtained from 710 bulls (body weight: 419.3 ± 59.18 kg; age 298.1 ± 33.10 d) enrolled in the University of Georgia’s and University of Tennessee’s bull evaluation programs, which included two locations in Georgia (Tifton and Calhoun; n = 520) and one location in Tennessee (Spring Hill; n = 190). Only Angus and SimAngus bulls consigned to the programs between 2019 and 2023 were included in this study. Data describing the population of bulls utilized in this study is summarized in Table 1. Upon arrival, bulls received broad-spectrum vaccines against clostridial (Caliber 7; Boehringer Ingelheim Animal Health USA Inc., Duluth, GA), respiratory, and reproductive pathogens (Express 10; Boehringer Ingelheim Animal Health USA Inc.), as well as endo- and ectoparasite control. Vaccine boosters were administered according to the manufacturer’s recommendation. Bulls were exposed to a 21-d adaption period for locations in Georgia and a 14-d adaptation period for bulls in Tennessee before the beginning of the performance test. The length of the performance test was 112 d for the years 2019 and 2020 and 84 d for the years 2021, 2022, and 2023 for Georgia locations. The length of the performance test was 84 d for Tennessee location. Within each year and location, all bulls were exposed to the same diets during both adaptation and performance evaluation periods. Diets consisted of a concentrated designed to optimize growth and development and ad libitum bermudagrass (*Cynodon dactylon*) hay or mixed of cool season forages (predominantly *Festuca arundinacea* and *Andropogon gerardii*) hay. Concentrate diet was developed based on each year’s commodity prices and designed to maximize growth performance of consigned bulls. Hence, minor variations in diet composition occurred depending on the year and location. Concentrate diet was also offered ad libitum in feed bins and separated from the hay.

**Table 1.** Descriptive statistics of 710 yearling beef bulls enrolled in bull evaluation programs at University of Georgia and University of Tennessee between 2019 and 2023^1^.

Item	Mean ± SD	Minimum	Maximum
Age, days	298.08	220.0	357.0
On test BW, kg	419.32 ± 59.18	265.81	589.67
On test WDA, kg/day	1.39 ± 0.15	0.98	1.80
On test hip height, cm	121.99 ± 4.80	110.49	135.26
Off test BW, kg	609.05 ± 61.22	429.10	795.15
Off test ADG, kg/day	2.01 ± 0.26	1.23	3.08
Off test WDA, kg	1.54 ± 0.12	1.19	1.90
Off test hip height, cm	130.64 ± 3.76	119.38	143.51
SCBF thickness, cm	0.88 ± 0.27	0.33	1.96
Ribeye area, cm^2^	98.41 ± 10.76	68.39	130.32
Intramuscular fat, %	4.67 ± 1.87	1.33	10.35

^1^BW: Body weight. WDA: Body weight per day of age. ADG: average daily gain during the test. SCBF: Subcutaneous backfat thickness estimated by ultrasonography. On test variables were collected at the beginning of the performance evaluation, whereas off test variables were collected at the end of the performance evaluation.

### Data Collection

Body weight was recorded twice within a 24-h interval at the beginning and end of the performance test. Hip height and weight per day of age (WDA) were assessed on day 0 and day 84 or 112, depending on the year. Weight per day of age was calculated following the guidelines of the Beef Improvement Federation. Final body weight assessment was utilized to estimate average daily gain (ADG), which was calculated by dividing the difference between final and initial body weight by number of days on test. Carcass ultrasonography was performed to evaluate ribeye area (REA), SCBF, and intramuscular fat (IMF; Detweiler et al., 2019). Furthermore, breeding soundness examinations (BSE) were performed within 40 d after carcass ultrasonography according to the Society of Theriogenology guidelines (Hopkins and Spitzer, 1997). Semen was collected via electroejaculation and motility was estimated subjectively as percentage of progressive motile sperm, followed by a categorical motility classification. Bulls were classified as having poor, fair, good, and very good motility when the subjectively estimated percentage of progressively motile sperm were ≤ 30, 31 to 49, 50 to 69, and ≥ 70%, respectively. Sperm morphology was estimated after eosin-nigrosin staining. In the present study, sperm head abnormalities were classified as primary morphology defects, whereas sperm tail and midpiece abnormalities were classified as secondary morphology defects (Barth and Oko, 1989). Breeding soundness examination records from bulls classified as unsatisfactory potential breeders or deferred for reasons unrelated to semen quality were not utilized in this study (n = 88). Exclusion criteria consisted of the presence of leukocytes in the ejaculate, inadequate scrotal circumference, abnormal seminal vesicle during rectal palpation, cryptorchidism, and suboptimal structural soundness (Hopkins and Spitzer, 1997; Koziol and Armstrong, 2018).

To evaluate the relationship between SCBF and fertility variables, bulls were categorized into three cohorts based on SCBF estimates using two distinct thresholds for SCBF. Within each location and year, bulls were retrospectively ranked based on their SCBF and classified into three groups: the top 10% (TOP10; n = 71), middle 80% (MID80; n = 569), and bottom 10% (BTM10; n = 70) for SCBF. To further explore the relationship between SCBF and fertility, bulls were also categorized into the top 20% (TOP20; n = 153), middle 60% (MID60; n = 419), and bottom 20% (BTM20; n = 138) based on SCBF.

### Statistical Analysis

All statistical analyses were conducted using the SAS statistical package (version 9.4, SAS Institute Inc., Cary, NC, USA) and bull was considered the experimental unit. Continuous response variables were analyzed using the MIXED procedure, whereas binary response variables were analyzed using the GLIMMIX procedure. Normality of continuous response variables were assessed using the Shapiro-Wilk normality test and all variables met the assumptions of normality. Models utilized to analyse continuous response variables related to performance (body weight, ADG, WDA, hip height, scrotal circumference, and carcass characteristics) included the fixed effect of SCBF classification using one of the two alternative thresholds while following a Gaussian distribution. Models utilized to analyze response variables associated with sperm cell morphology during the breeding soundness examination (percentage of normal cells, percentage of cells with primary and secondary abnormalities) were analyzed using the fixed effects of SCBF classifications with one of the two alternative thresholds while following a beta distribution.

Models utilized to analyze the impact of SCBF on the percentage of bulls with fair or poor motility and the percentage of bulls that were not classified as satisfactory breeders included the fixed effects of SCBF classification with one of the two alternative thresholds. However, these response variables were modeled using a binary distribution with a logit link function. Odds ratio and their 95% confidence intervals were also evaluated to estimate the effect of backfat thickness classifications on the odds of failing the breeding soundness examination relative to a reference group. Cohorts MID80 and MID60 were considered the reference groups for these analyses.

To further investigate the relationship between SCBF and the probability of bulls failing the BSE, a generalized linear mixed model was fitted using the GLIMMIX procedure with the Laplace approximation method. The model followed a binomial distribution with a logit link function, initially including linear, quadratic, and cubic terms for SCBF. Predicted failure probabilities were generated for statistically significant effects from this model and plotted to visually assess the relationship between SCBF and BSE failure probability.

All statistical models included the random effect of location and year. The statistical analysis reported statistical significance at *P* ≤ 0.05, and 0.05 < *P *≤ 0.10 was considered a statistical tendency. Data is reported as mean ± SEM unless otherwise stated.

## RESULTS

### Impact of Bull Subcutaneous Backfat Thickness Classification on Bull Performance and Carcass Characteristics

Data summarizing bull performance according to SCBF classification using a 10% threshold is displayed in Table 2. TOP10 bulls had greater body weight at the beginning of the performance test compared with BTM10 and MID80 bulls (*P* < 0.01), which also differed between them (*P *= 0.03). The same results were observed for WDA at the beginning of the test (*P *< 0.01), as well as body weight (*P* ≤ 0.01) and WDA (*P *< 0.01) at the end of the performance evaluation. Age at the beginning of the test (*P *= 0.17) and average daily gain (ADG; *P *= 0.29) during the test did not differ between SCBF classifications. Bulls classified as TOP10 for SCBF had the greatest (*P *< 0.01) SCBF compared with MID80 and BTM10 bulls, whereas MID80 bulls had greater (*P *< 0.01) SCBF compared with BTM10 bulls. Similar results were observed for IMF and REA, where TOP10 bulls had greater IMF (*P *< 0.01) and REA (*P *< 0.01) compared with MID80 and BTM10 bulls, and MID80 bulls had greater IMF (*P *< 0.01) and REA (*P *< 0.01) compared with BTM10 bulls. Hip height did not differ between SCBF classifications in the beginning (*P *= 0.64) and at the end of the performance test (*P *= 0.20; Table 2).

**Table 2.** Performance characteristics of yearling beef bulls based on subcutaneous backfat thickness classifications (SCBF) using a 10% threshold^1^.

Item	Bottom 10%	Middle 80%	Top 10%	*P*-value
n	71	569	70	-
Age, days	306.56 ± 15.56	301.33 ± 15.30	304.98 ± 15.56	0.17
On test BW, kg	408.56 ± 21.75^a^	422.29 ± 21.02^b^	447.57 ± 21.76^c^	*<* 0.01
On test WDA, kg/day	1.31 ± 0.03^a^	1.37 ± 0.03^b^	1.45 ± 0.03^c^	*<* 0.01
On test hip height, cm	122.50 ± 2.27	122.15 ± 2.24	122.43 ± 2.27	0.64
Off test BW, kg	595.19 ± 16.66^a^	613.26 ± 15.44^b^	641.98 ± 16.68^c^	*<* 0.01
Off test ADG, kg/day	1.99 ± 0.05	2.01 ± 0.04	2.05 ± 0.05	0.29
Off test WDA, kg/day	1.47 ± 0.03^a^	1.53 ± 0.02^b^	1.59 ± 0.03^c^	*<* 0.01
Off test hip height, cm	131.65 ± 1.16	130.91 ± 1.10	130.81 ± 1.63	0.20
SCBF thickness, cm	0.52 ± 0.08^a^	0.86 ± 0.08^b^	1.32 ± 0.08^c^	*<* 0.01
Ribeye area, cm^2^	94.32 ± 4.02^a^	97.61 ± 3.89^b^	101.42 ± 4.02^c^	*<* 0.01
Intramuscular fat, %	3.84 ± 1.02^a^	4.47 ± 1.01^b^	5.23 ± 1.02^c^	*<* 0.01
Scrotal circumference, cm	37.88 ± 0.33	38.36 ± 0.14	38.29 ± 0.33	0.35

^1^Subcutaneous backfat (SCBF) thickness was estimated via ultrasonography. Bulls were ranked based on SCBF and classified into the top 10%, middle 80%, and bottom 10% for SCBF. BW: Body weight. WDA: Body weight per day of age. ADG: average daily gain during the test. On test variables were collected at the beginning of the performance evaluation, whereas off test variables were collected at the end of the performance evaluation. ^a,b,c^ Uncommon superscript within row represent statistical differences between SCBF classification (*P *≤ 0.05).

Performance data according to SCBF classification using a 20% threshold is summarized in Table 3. Initial body weight differed between TOP20, MID60, and BTM20 bulls (*P *< 0.01 for all comparisons). Body weight at the end of the performance test was also greater (*P *< 0.01) in TOP20 bulls compared with MID60 and BTM20 bulls and greater (*P *< 0.01) in MID60 compared with BTM20 bulls. There were no differences in age at the beginning of the test and ADG during the test between bulls with different SCBF classifications (*P *≥ 0.86). Bulls classified as TOP20 had the greatest (*P *< 0.01) SCBF compared with MID60 and BTM20, and MID60 bulls had greater (*P *< 0.01) SCBF compared with BTM20 bulls. Similar results were observed for the percentage of IMF and REA, with TOP20 bulls having greater IMF (*P *< 0.01) and REA (*P *< 0.01) compared with MID60 and BTM20 bulls. Moreover, MID60 bulls had greater IMF (*P *< 0.01) and REA (*P *< 0.01) compared with BTM20 bulls. Hip height did not differ between SCBF classifications in the beginning (*P *= 0.77) and at the end of the performance test (*P *= 0.11; Table 3).

**Table 3.** Performance characteristics of yearling beef bulls based on subcutaneous backfat thickness classifications (SCBF) using a 20% threshold^1^.

Item	Bottom 20%	Middle 60%	Top 20%	*P*-value
n	153	419	138	-
Age, days	303.26 ± 15.43	301.94 ± 15.34	301.97 ± 15.44	0.86
On test BW, kg	406.11 ± 21.36^a^	424.28 ± 21.11^b^	439.95 ± 21.40^c^	*<* 0.01
On test WDA, kg/day	1.32 ± 0.03^a^	1.38 ± 0.03^b^	1.44 ± 0.03^c^	*<* 0.01
On test hip height, cm	122.39 ± 2.25	122.14 ± 2.24	122.22 ± 2.25	0.77
Off test BW, kg	597.53 ± 15.91^a^	614.87 ± 15.47^b^	630.85 ± 15.97^c^	*<* 0.01
Off test ADG, kg/day	2.01 ± 0.05	2.01 ± 0.05	2.01 ± 0.05	0.95
Off test WDA, kg/day	1.49 ± 0.03^a^	1.53 ± 0.03^b^	1.58 ± 0.03^c^	*<* 0.01
Off test hip height, cm	131.49 ± 1.12	130.87 ± 1.10	130.73 ± 1.13	0.11
SCBF thickness, cm	0.59 ± 0.08^a^	0.86 ± 0.08^b^	1.23 ± 0.08^c^	*<* 0.01
Ribeye area, cm^2^	94.47 ± 3.90^a^	98.00 ± 3.86^b^	100.14 ± 3.91^c^	*<* 0.01
Intramuscular fat, %	3.90 ± 1.01^a^	4.48 ± 1.01^b^	5.15 ± 1.02^c^	*<* 0.01
Scrotal circumference, cm	38.14 ± 0.24	38.38 ± 0.17	38.26 ± 0.25	0.61

^1^Subcutaneous backfat thickness was estimated via ultrasonography. Bulls were ranked based on SCBF and classified into the top 20%, middle 60%, and bottom 10% for SCBF. BW: Body weight. WDA: Body weight per day of age. ADG: average daily gain during the test. On test variables were collected at the beginning of the performance evaluation, whereas off test variables were collected at the end of the performance evaluation. ^a,b,c^ Uncommon superscript within row represent statistical differences between SCBF classification (*P *≤ 0.05).

### Subcutaneous Backfat Thickness and Semen Analyses

**Figure 1.**
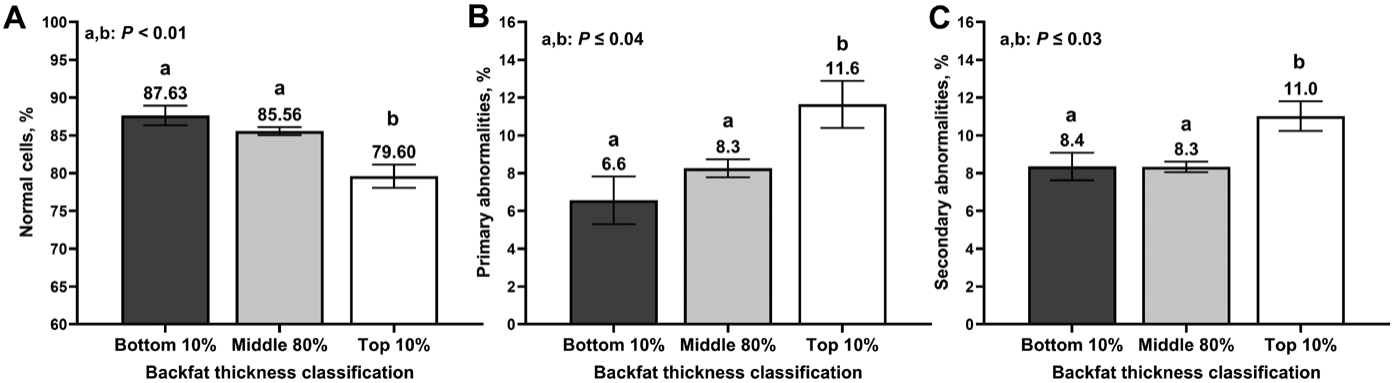
Sperm morphology outcomes of yearling beef bulls (n = 710) according to subcutaneous backfat classification. Subcutaneous backfat thickness was estimated via ultrasonography and bulls were ranked and classified into the top 10% (n = 70), middle 80% (n = 569), and bottom 10% (n = 71) for subcutaneous backfat thickness. A breeding soundness examination was performed, and sperm morphology was estimated following the guidelines of the Society of Theriogenology (Hopkins and Spitzer, 1997).

The percentage of bulls classified as having poor or fair motility was not impacted by SCBF thickness classifications when using both 10% (*P *= 0.37; TOP10: 21.7 ± 7.82%; MID80 17.9 ± 5.42%; and BTM10: 12.6 ± 5.54%) or 20% (*P *= 0.60; TOP20: 17.8 ± 6.13%; MID60 18.7 ± 5.69%; and BTM20: 15.0 ± 5.35%) threshold. There were also no effects of SCBF on scrotal circumference based on a 10% threshold (*P = *0.35; Table 2). The percentage of morphologically normal sperm cells was greater (*P *< 0.01) in BTM10 and MID80bulls compared with TOP10 bulls (Figure 1A); however, there were no differences between BTM10 and MID80 bulls (*P *= 0.30). There was also an effect of SCBF on sperm primary abnormalities (*P = *0.02; Figure 1B), where TOP10 had a greater percentage of sperm cells with primary abnormality compared with MID80 (*P* = 0.04) BTM10 bulls (*P* = 0.04). No differences in the percentage of primary abnormalities were observed between BTM10 and MID80 bulls (*P* = 0.49). There was also an effect of SCBF classification on the percentage of secondary abnormalities (*P *= 0.01; Figure 1C), where TOP10 bulls had a greater percentage of secondary abnormalities compared with MID80 (*P *< 0.01) and BTM10 bulls (*P *= 0.03), which were not different among them (*P *= 0.97).

**Figure 2.**
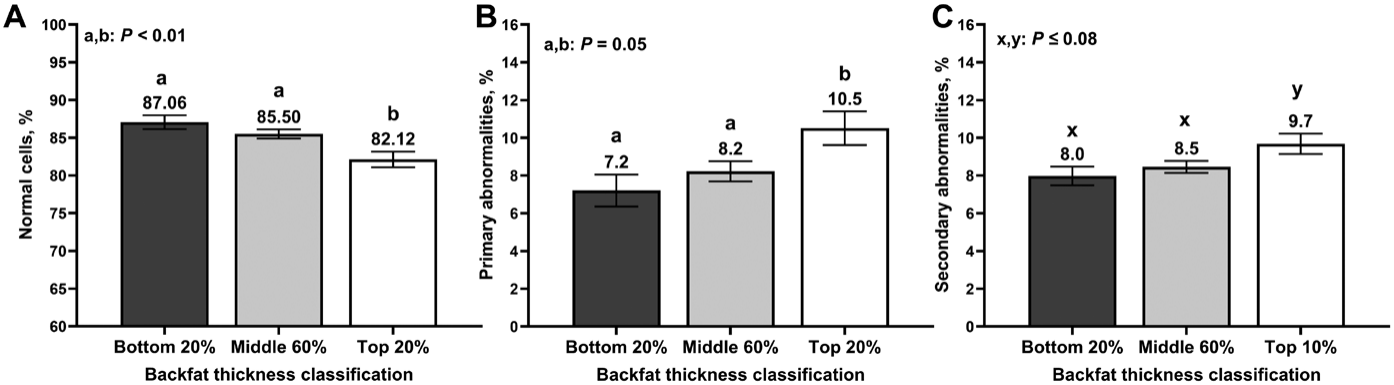
Sperm morphology outcomes of yearling beef bulls (n = 710) according to subcutaneous backfat classification. Subcutaneous backfat thickness was estimated via ultrasonography and bulls were ranked and classified into the top 20% (n = 138), middle 60% (n = 419), and bottom 10% (n = 153) for subcutaneous backfat thickness. A breeding soundness examination was performed, and sperm morphology was estimated following the guidelines of the Society of Theriogenology (Hopkins and Spitzer, 1997).

There were no differences between SCBF classification for scrotal circumference based on a 20% threshold (*P *= 0.61; Table 3). There was an effect (*P *< 0.01) of SCBF classification on the percentage of normal sperm cells, where TOP20 bulls had a decreased percentage of sperm cells classified as morphologically normal compared with MID60 (*P *< 0.01) and BTM20 bulls (*P *< 0.01; Figure 2A). There were no differences in the percentage of normal cells between MID60 and BTM20 (*P *= 0.31). TOP20 bulls also had a greater percentage of sperm cells with primary abnormalities compared with MID60 bulls (*P =* 0.04) and BTM20 (*P *= 0.02), which were similar among them (*P *= 0.35; Figure 2B). There was also a tendency (*P* = 0.10) for an effect of SCBF on the percentage of sperm cells with secondary abnormalities, where TOP20 bulls had greater (*P *= 0.04) percentage of secondary abnormalities compared with BTM20 and tended (*P *= 0.08) to have a greater percentage of secondary abnormalities compared with MID60 bulls (Figure 2C). There were no differences between MID60 and BTM20 bulls for secondary abnormalities (*P *= 0.47).

### Breeding Soundness Examination Results

**Figure 3.**
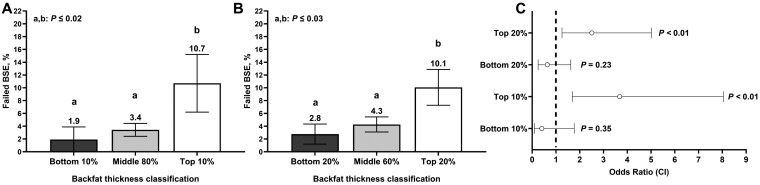
Breeding soundness examination (BSE) results of yearling beef bulls (n = 710) according to subcutaneous backfat classifications. Subcutaneous backfat thickness was estimated via ultrasonography and bulls were ranked and classified using two different thresholds: 1) Bulls were divided into the top 10% (n = 70), middle 80% (n = 569), and bottom 10% (n = 71) for subcutaneous backfat thickness (**Panel A**); 2) Bulls were divided into the top 20% (n = 138), middle 60% (n = 419), and bottom 10% (n = 153) for subcutaneous backfat thickness (**Panel B**). Bulls were considered to have failed the BSE when they were classified as deferred (Hopkins and Sptizer, 1997). **Panel C** represents odds ratio and 95% confidence intervals for BSE failure according to subcutaneous backfat thickness classification. Bulls classified in the middle 60% and 80% of the population served as reference for the odds ratio analysis.

**Figure 4.**
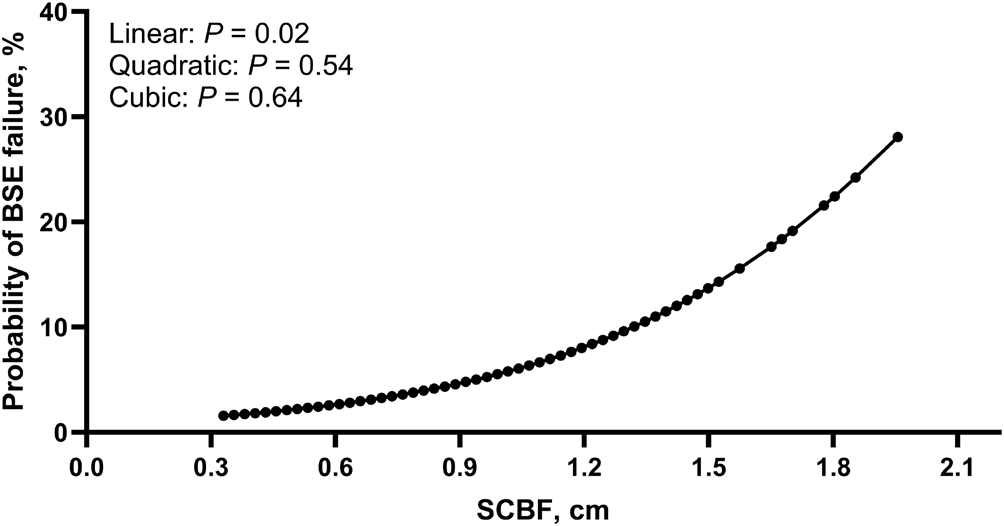
Relationship between subcutaneous backfat thickness (SCBF) and probability of breeding soundness examination (BSE) failure in young beef bulls.

A total of 137 bulls failed their BSE, which represented 17.1% (137/798) of the bulls enrolled in the bull development programs described herein. Moreover, a total of 49 bulls failed their BSE based solely on semen motility and morphology outcomes, which represented 6.9% (49/710) of the population enrolled in this study. A greater percentage of TOP10 bulls failed the BSE based on the semen analysis compared with MID80 and BTM10 bulls (*P *< 0.01); however, no differences were observed between MID80 and BTM10 (*P *= 0.23; Figure 3A). A decreased percentage of TOP20 bulls were classified as satisfactory potential breeders compared with both MID60 (*P *< 0.01) and BTM20 bulls (*P *< 0.01), which were similar among them (*P* = 0.35; Figure 3B). Based on the odds ratio analyses, bulls in the TOP10 had 3.68 times lower odds of being classified as a satisfactory potential breeder compared with MID80 bulls (*P *< 0.01; Figure 3C). In addition, bulls in the TOP20 had 2.51 times lower odds of being classified as satisfactory potential breeders compared with MID60 bulls (*P *< 0.01; Figure 3C). Finally, there was a positive linear (*P* = 0.02) effect of SCBF on the probability of BSE failure where a progressive increase in BSE failure probability was observed with increasing SCBF (Figure 4). There were no quadratic or cubic effects (P ≤ 0.54)

## DISCUSSION

Although the negative relationship between overnutrition and semen quality has been previously described, most of the literature on this topic is based on studies where bulls were experimentally fed to achieve divergent rates of ADG (Coulter and Kozub, 1984; Coulter et al., 1987, 1997). Alternatively, the present research used an observational study design to investigate associations between naturally occurring variations in adiposity and semen parameters during industry-standard BSE. We hypothesized that bulls with excessive adiposity, indirectly assessed based on SCBF, would exhibit decreased semen quality compared to bulls with adequate SCBF, despite all bulls being exposed to the same diet. Results reported in this study support our hypothesis and indicate a negative relationship between SCBF and semen quality in yearling beef bulls.

Beef bulls are commonly developed on a higher plane of nutrition to maximize body weight gain and highlight their genetic potential for post-weaning growth (Oosthuizen et al., 2018). Similarly, bulls in the present study were exposed to a diet that allowed for a mean ADG of 2.01 kg, which is similar to that observed in feedlot steers (Marques et al., 2017; Pancini et al., 2020) and other bull development programs (Oosthuizen et al., 2018). Mean SCBF was 0.88 cm and ranged from 0.33 to 1.96 cm. This variation allowed us to explore the impact of increased adiposity on semen parameters using a relatively large number of BSE records, which is uncommon in bull fertility studies. Moreover, the levels of adiposity observed in bulls that were in the extreme population for SCBF (TOP10 and TOP20 bulls) resemble those of studies where bulls were experimentally fed to induce an overnutrition phenotype (Coulter et al., 1984; Seekford et al., 2023), despite all bulls in the present study being maintained in the same diet for at least 105 d. This is an important distinction that indicates an impact of adiposity on semen quality rather than a direct impact of the diet on spermatogenesis or sperm development in the epididymis. High energy diets can elicit acidosis, which has been shown to decrease circulating concentrations of FSH and testosterone, disrupt spermatogenesis, and increase the percentage of sperm morphology defects (Callaghan et al., 2016).

Increased SCBF was associated with a decreased percentage of sperm cells classified as morphologically normal. These results were observed regardless of the thresholds used to create different SCBF cohorts. Bulls in the extreme of the population for SCBF (TOP10 and TOP20 bulls) had increased primary abnormalities compared with the other cohorts. Similarly, TOP10 bulls had increased secondary abnormalities compared with MID80 and BTM10, whereas TOP20 bulls tended to have increased secondary abnormalities compared with MID60 and BTM20. Therefore, the decrease in the percentage of morphologically normal cells occurred as a result of differences in both primary and secondary abnormalities. There were no differences between cohorts in the percentage of bulls classified as having poor or fair sperm motility. Although some studies reported that bull overnutrition reduced sperm motility (Coulter et al., 1997), others have also reported no differences (Callaghan et al., 2016; Seekford et al., 2023).

This lack of differences in sperm motility estimates could be explained by the subjective nature of motility evaluations in the field or the fact that motility was reported as a categorical response variable, limiting our ability to statistically detect differences between cohorts.

Bulls classified as TOP10 and TOP20 were heavier throughout the performance evaluation period when compared with their respective cohorts. Moreover, differences in carcass characteristics between SCBF classifications were not limited to SCBF. Bulls in TOP10 and TOP20 had greater IMF and REA compared with their respective cohorts, even though there were no differences in age between SCBF classifications. Interestingly, although differences in body weight and carcass composition were clearly present between SCBF classifications, growth performance during the test was not different. These results suggest that the improved semen quality observed in leaner bulls was not accompanied by a detrimental effect on average daily gain.

Impaired testicular thermoregulation has been proposed as a potential cause of sperm morphological abnormalities in overnutrition models. It is well-established that testicular temperature needs to be 4 to 5 °C below body core temperature to sustain spermatogenesis, and experimentally increasing testicular and epididymal temperatures through scrotal insulation increases sperm morphological abnormalities (Vogler et al., 1991, 1993; Kastelic et al., 1996), deteriorates acrosome integrity, and increases sperm oxidative stress (Barth and Bowman, 1994; Kastelic et al., 2018; Ferrer et al., 2020). Moreover, SCBF is positively correlated with fat accumulation in the vascular cone region (Brito et al., 2012). Kastelic et al., (1996) insulated the neck region of the scrotum as a model to mimic nutritionally induced fat accumulation in the proximal portion of the scrotum. Insulation of scrotal neck region resulted in increased intratesticular temperature and increased percentage of sperm morphology defects, particularly in spermatozoa within the epididymis or at the acrosome phase during insulation (Kastelic et al., 1996). Similar consequences to semen quality were observed when yearling beef bulls were fed a high concentrate diet to achieve high rates of ADG for 168 d. Coulter et al. (1997) observed an increase in scrotal circumference and a decrease in scrotal surface temperature gradient between the proximal and distal portions of the scrotum in bulls fed a high-energy diet. This lack of temperature gradient between the proximal and distal regions of the scrotum could reflect a suboptimal regulation of testicular temperature. These changes were also associated with a decrease in sperm motility and a decrease in the percentage of morphologically normal sperm in bulls fed a high-energy diet (Coulter et al., 1997). Others also reported increased scrotal circumference and a greater percentage of morphological abnormalities in bulls exposed to high-energy diets for a prolonged period of time (Mwansa and Makarechian, 1991). In the present study, there were no differences in scrotal circumference between SCBF classifications regardless of the threshold utilized in the analysis. Moreover, there was no correlation between SCBF and scrotal circumference (data not reported). This lack of association between SCBF and scrotal circumference has been previously reported when bulls were fed a common diet (Brito et al., 2012). Based on the limited information on testicular thermoregulation in the present study, it is unclear whether bulls in the TOP10 or TOP20 cohorts experienced suboptimal testicular thermoregulation. Studies in humans and rodents have shown that diet-induced obesity increases systemic inflammation, resulting in increased sperm oxidative stress, chromatin damage, and epigenetic modifications that collectively decrease male fertility (Barbagallo et al., 2021). In cattle, changes in plane of nutrition have been shown to alter seminal plasma cytokine profile (Harrison et al., 2023). Hence, systemic and local inflammation might also contribute to changes in semen characteristics in individuals with increased adiposity. Further research is required to better describe the impact of diet on local and systemic inflammatory markers associated with excessive adiposity in bulls.

Overall BSE failure rate was 17% (137/798) in bulls enrolled in the bull development programs described herein. Eighty-eight of these bulls failed because of the presence of leukocytes in the ejaculate, inadequate scrotal circumference, abnormal seminal vesicle during rectal palpation, cryptorchidism, and suboptimal structural soundness. Because these BSE failures were associated not associated with sperm motility or morphology, they were excluded from the dataset reported herein. A total of 49 bulls failed their BSE for reasons related to semen evaluation outputs, which represented 6.9% (49/710) of the BSE performed in this study. Therefore, the prevalence and cause of BSE failure reported herein resembles reports by others (Barth and Waldner, 2002; Roberts et al., 2018), indicating that the present study is representative of what is commonly observed in the beef industry.

Percentage of bulls failing the BSE was influenced by SCBF cohort. Although differences in the percentage of morphologically normal sperm were only slightly (< 10%) increased in TOP10 and TOP20 bulls compared with the other cohorts, a greater percentage of TOP10 and TOP20 bulls were classified as deferred when compared to their respective cohorts. In fact, TOP10 bulls had 3.68 times greater odds of being classified as deferred potential breeders during the BSE compared with MID80 These results were also observed when using a 20% threshold. Bulls in the TOP20 had 2.51 times greater odds of receiving a differed diagnosis during the BSE. Because of the continuous rather than categorical nature of SCBF, the present dataset allowed us to model the relationship between SCBF and the probability of bulls failing their BSE. Our results indicate a linear increase in the probability of BSE failure as SCBF increased. Notably, the predicted failure probability becomes clearly elevated at approximately 1.4 cm SCBF, suggesting this value may represent a biologically meaningful threshold beyond which bull fertility is increasingly compromised. Few studies explored the relationship between normal variation in adiposity and BSE results. Barth and Waldner (2002) retrospectively evaluated BSE examination records in structurally sound bulls according to bull body condition scores using a 1 to 5 scale. Fewer bulls with body condition scores < 2.5 or ≥ 4 were classified as satisfactory breeders compared with bulls of body condition scores between 2.5 and 3.5. Although the latter study used BSE records from multiple herds and a subjective estimate of adiposity, results indicated that bulls with increased adiposity were more likely to fail a breeding soundness examination. Barth and Waldner (2002) utilized only bulls that were at least 16 m of age at the time of BSE, highlighting the importance and novelty of the present study, which focused on young beef bulls. To our knowledge, no studies have specifically examined the relationship between variation in beef bull adiposity, objectively quantified via ultrasonography, and the proportion of bulls failing the BSE in populations of bulls managed under identical environmental, nutritional, and management conditions. These findings emphasize a consistent response observed across different methodologies and studies, strengthening the evidence that excessive adiposity negatively impacts fertility in beef bulls.

In summary, increased adiposity in young beef bulls exposed to the same diet was associated with a greater percentage of sperm with morphological abnormalities during industry-standard BSE. These differences in sperm morphology were observed for both primary and secondary abnormalities. Although mean differences in the percentage of morphologically normal cells were subtle between SCBF cohorts, these differences in sperm morphology were associated with a greater percentage of bulls with excessive SCBF receiving a differed classification in their first BSE. Interestingly, while bulls with excessive SCBF were heavier during the performance evaluation, similar ADG was observed between bulls with different SCBF. These results suggest that the improved semen quality observed in leaner bulls was not associated with decreased performance.
